# A Multicenter Non‐Inferiority Randomized Controlled Trial of Sentinel Lymph Node Biopsy in Early Breast Cancer: Conventional Dual Tracer Versus Superparamagnetic Iron Oxide

**DOI:** 10.1002/wjs.70498

**Published:** 2026-07-31

**Authors:** Chiu Hoi Woon Tiffany, Ray Ka Wai Hung, Kong Wai Chung Angela, Kwok Sin Man, Sheung Robin Lok Man, Violet Yee Kei Tsoi

**Affiliations:** ^1^ Department of Surgery The Chinese University of Hong Kong Hong Kong China; ^2^ Department of Surgery North District Hospital Hong Kong China; ^3^ Department of Surgery Princess Margaret Hospital Hong Kong China

**Keywords:** breast cancer, breast surgery, sentinel lymph node, superparamagnetic iron oxide

## Abstract

**Background:**

The gold standard for detecting sentinel lymph node (SLN) in early breast cancer is combined use of radioisotope and blue dye. A newer agent, superparamagnetic iron oxide (SPIO), was developed to address the challenges faced with the traditional method. This study aims to compare the detection rate of SLN biopsy and show the non‐inferiority of SPIO.

**Methods:**

From March 2021 to April 2024, patients with clinically and radiologically node‐negative breast cancer were randomized to receive SPIO‐only injection or dual tracer for SLN localization. The primary endpoint was the SLN identification rate in both arms.

**Results:**

148 patients were in the SPIO group with 153 SLN procedures performed. SLN localization failed in one patient. The identification rate was 99.3% (95% CI: 95.8–99.9). 149 patients were in the dual tracer group with 156 SLN procedures done. The identification rate was 100% (95% CI: 97.0–99.9). The absolute difference was 0.7% (95% CI: −1.2 to 2.7).

**Conclusion:**

Our results suggest that SPIO, as a single agent, used in SLN biopsy for early breast cancer is non‐inferior to the dual tracer method. It can serve as a good alternative to radioactive isotope and blue dye.

**Trial Registration:**

CUHK CREC Ref No. 2019.677‐T (https://www.crec.cuhk.edu.hk/)

## Graphical abstract

The gold standard for detecting sentinel lymph node (SLN) in early breast cancer is combined use of radioisotope and blue dye. A newer agent, superparamagnetic iron oxide (SPIO), was developed to address the challenges faced with the traditional method. This study aims to compare the detection rate of SLN biopsy and show the non‐inferiority of SPIO. Our results suggest that SPIO, as a single agent, used in SLN biopsy for early breast cancer is non‐inferior to the dual tracer method. It can serve as a good alternative to radioactive isotope and blue dye.

## 1 Introduction

Sentinel lymph node biopsy (SLNB) is the current standard of care for staging the axillary status in clinically node‐negative breast cancer patients. With the introduction of SLNB, it has led to a decrease in patient morbidity from axillary dissection, including lymphedema, reduced shoulder mobility, and chronic pain [1].

In SLNB, a tracer is injected into the subcutaneous tissue of the breast and then travels along the lymphatic channels and accumulates in the sentinel lymph node (SLN), mimicking the metastatic pathway of tumor cells. Radioisotope colloid and blue dye are the two most commonly used tracers, either alone or in combination. They are considered the gold standard with the highest detection rate and low false negative rate [2]. However, radioactive tracers come with drawbacks. The radio‐labeled tracer (technetium‐99m) carries radiation exposure, has a relatively short half‐life (6 h), limits surgery scheduling, and poses logistical challenges in the handling and transporting of the tracer. Worldwide shortages of technetium‐99m have also been predicted [3]. Blue dye also carries an adverse risk reaction of up to 0.9% [4] and frequently obscures the surgical field. As a result, novel agents have been explored to replace the conventional method to overcome these challenges. Superparamagnetic iron oxide (SPIO) is one of these techniques, which has shown promising results.

SPIO can be injected at least 20 min before surgery and pre‐operatively up to 30 days before the procedure and poses no radiation hazard [3, 5, 6, 7]. It is a blackish‐brown suspension of carboxydextran‐coated SPIO particles. It is around 60 nm in size, similar to that of radioisotope tracers, which allows efficient uptake into lymphatic channels and subsequent retention in the SLN [8]. It also stains the SLN brown, which also helps visual identification intraoperatively. Multiple feasibility and non‐inferiority studies have been published, showing the success of SPIO and high concordance with the dual tracer method [5, 6, 7, 8, 9, 10]. Two meta‐analyses have also demonstrated the high SLN identification rate of SPIO comparable to dual tracer [11, 12]. The detection rate with dual tracer has been reported to be up to 99% [3]. Majority of these trials administered SPIO concurrently with blue dye or radioisotope, potentially overestimating its standalone performance due to synergy bias. Data on SPIO used as a single agent remain relatively limited, particularly in Asian populations. The superiority of SPIO would be difficult to prove, hence, we performed a non‐inferiority trial comparing SPIO as a single agent and dual tracer.

## 2 Patients and Methods

### 2.1 Study Design

This is a multi‐center, prospective, randomized non‐inferiority randomized controlled trial carried out from March 2021 to April 2024 at two major breast centers in Hong Kong. This study was approved by the local ethics committee in each center. Written informed consent was obtained prior to randomization.

### 2.2 Participants

Patients diagnosed with breast cancer (including ductal carcinoma in situ) with clinically and radiologically node‐negative disease, were recruited. Exclusion criteria were history of ipsilateral axillary surgery or radiation, known hypersensitivity to iron or dextran compounds or blue dye, known iron‐overload disease, presence of pacemakers or implantable devices on the chest wall, and non‐palpable breast cancer requiring simultaneous localization.

Patients were assigned into 2 arms in a 1:1 ratio by a computer‐generated sequence in block randomization with block size 20, after obtaining consent by a breast surgeon or nurse. Patients and surgeons were not blinded to the procedure.

### 2.3 Surgical Technique

In the dual‐tracer arm, patients received technetium‐99m (Tc‐99m)‐labeled nanocolloid injected periareolarly in the nuclear medicine department, either the day before or on the morning of surgery. Following induction of general anesthesia, 1 mL of methylene blue dye was injected periareolarly, followed by 5 min of breast massage. Transcutaneous gamma‐probe counts were obtained prior to incision. Sentinel lymph node biopsy (SLNB) was performed via the mastectomy incision or a separate axillary incision, at the operating surgeon's discretion. All nodes demonstrating blue color or elevated radioactivity (hot nodes) were excised. Excision was considered complete when the residual axillary count was < 10% of the highest ex vivo count and no additional blue nodes were identified. In the SPIO arm, 1–2 mL of Magtrace (Endomagnetics Ltd., Cambridge, UK) was injected periareolarly, with or without massage, at least 20 min prior to incision. SLNB was performed using the handheld SentiMag (Endomagnetics Ltd., Cambridge, UK) magnetometer probe. Non‐metallic instruments were employed when necessary to minimize probe interference. Incision site and approach were determined by the primary procedure and surgeon preference. Sentinel nodes were identified by magnetic signal detection and/or visible brown staining. Nodes were excised until the axillary magnetic count fell to < 10% of the highest ex vivo count and no additional brown nodes were identified.

In both arms, clinically suspicious nodes lacking tracer signal or color were excised at the surgeon's judgment but were not classified as tracer‐identified sentinel nodes. If no sentinel nodes were detected by tracer criteria, then level I–II axillary lymph node dissection was performed. All excised nodes underwent standard histopathological evaluation. Patients were followed up post‐operatively according to the standard protocol of each center.

### 2.4 Study Objectives

The primary endpoint was the rate of successful identification of sentinel lymph nodes. Secondary endpoints were evaluated, including the number of sentinel lymph nodes identified, operation time, and possible adverse events.

### 2.5 Statistical Analysis

The identification rates of SLNB by SPIO and conventional dual tracer method were 99.3% and 98.6%, respectively [9]. With a one‐sided significant level of 0.025, a non‐inferior margin of 5%, and statistical power at 80%, the sample size was calculated to be 150 in each arm.

Data analysis was performed with Statistical Product and Service Solution Statistics 27. Categorical variables were summarized with frequency tables (percentages), and quantitative variables were expressed as median with interquartile range (IQR). Two groups were compared using chi‐square, Fischer exact test, and Mann‐Whitney *U* test when appropriate. Regression analysis was performed in subgroup analysis.

## 3 Results

**FIGURE 1:**
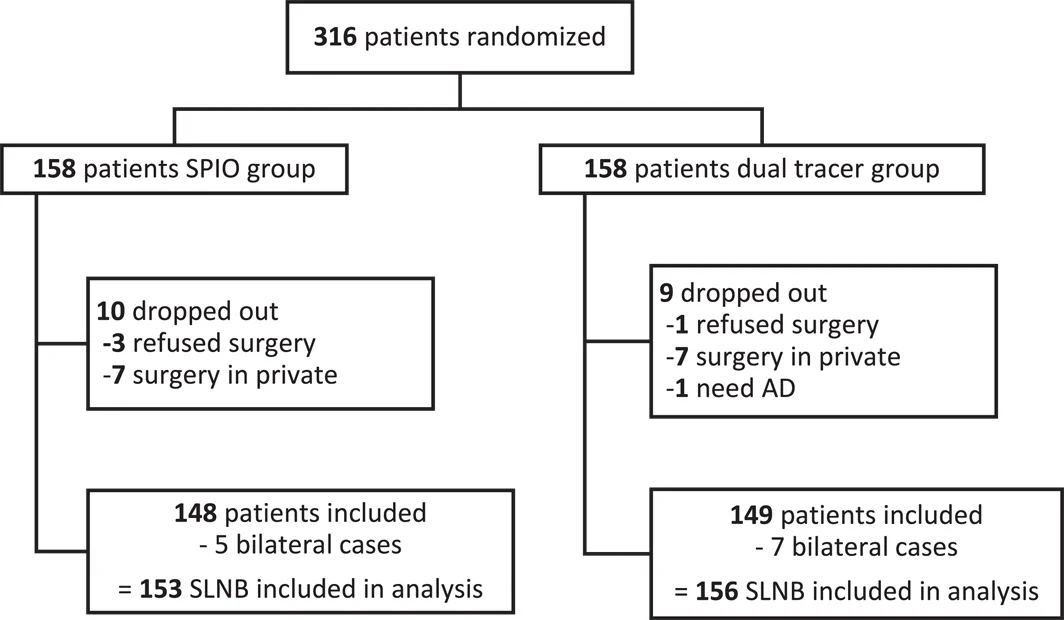
CONSORT diagram.

Between March 2021 and April 2024, 316 patients were randomized (Figure 1). After exclusions (10 in SPIO arm, 9 in dual‐tracer arm), 148 patients (153 SLNB procedures) were analyzed in the SPIO arm and 149 patients (156 SLNB procedures) in the dual‐tracer. 4 patients refused surgery after randomization, 1 patient was found to have lymph node metastasis preoperatively, and the remaining had earlier surgery in the private sector. A total of 156 SLNB were performed with dual tracer and 153 SLNB with Magtrace. 12 patients had bilateral cancers and underwent bilateral SLNB. In the dual tracer group, 4 patients had Magtrace injected for SLNB due to logistics difficulties for radioactive colloid arrangements. They were included in the control group according to intention to treat analysis.

Baseline characteristics of the two populations are shown in Table 1, which are comparable in both arms. There was 1 male patient. The median age was 64 years for both groups, with the majority of patients being menopausal. Median tumor size was similar, around 1.7–1.8 cm. Tumor type, nuclear grading, and biology were comparable, except for tumor location (*p* = 0.037). Most patients (∼ 80%) received mastectomy in both cohorts, with the final pathology showing predominantly T1 or T2 disease (> 70%).

**TABLE 1:** Baseline patient and tumor characteristics.

	SPIO group	Dual tracer group	*p* value
Number of patients	148	149	
Number of SLN	153	156	
Age, median (IQR)	64 (54–71)	63.5 (52–70)	0.185
BMI, median (IQR)	24.38 (21.9–27.3)	23.81 (21.4–26.6)	0.293
Tumor size on USG (cm), median (IQR)	1.7 (1.2–2.4)	1.8 (1.2–2.6)	0.573
Laterality			0.331
Left	70 (45.8%)	80 (51.6%)	
Right	83 (54.2%)	76 (48.7%)	
Presentation			0.466
Screening	18 (11.8%)	14 (9.0%)	
Palpable lump	98 (64.1%)	110 (70.5%)	
Others	37 (24.2%)	32 (20.5%)	
Menopaused	124 (81.0%)	118 (75.6%)	0.249
Female	152 (99.3%)	156 (100%)	0.312
Location of tumor			0.037
Upper outer quadrant	68 (44.4%)	63 (40.4%)	
Upper inner quadrant	19 (12.4%)	22 (14.1%)	
Lower inner quadrant	7 (4.6%)	5 (3.2%)	
Lower outer quadrant	14 (9.2%)	33 (21.2%)	
Central	45 (29.4%)	33 (21.2%)	
History of breast surgery			0.254
Yes	7 (4.6%)	12 (7.7%)	
No	146 (95.4%)	144 (92.3%)	
Tumor type			0.482
DCIS	34 (22.2%)	25 (16%)	
IDC	110 (71.9%)	119 (76.3%)	
ILC	5 (3.3%)	5 (3.2%)	
Others	4 (2.6%)	7 (4.5%)	
Nuclear grade			0.277
1	36 (23.5%)	29 (18.6%)	
2	56 (26.6%)	62 (39.7%)	
3	27 (17.6%)	40 (25.6%)	
Tumor Biology			0.098
HR+, HER2−	120 (78.4%)	105 (67.3%)	
HR+, HER2+	11 (7.2%)	18 (11.5%)	
HR−, HER2+	4 (2.6%)	11 (7.1%)	
HR−, HER2−	14 (9.2%)	13 (8.3%)	
Type of OT			0.602
Mastectomy	123 (80.4%)	122 (78.2%)	
BCT	18 (11.8%)	24 (15.4%)	
Reconstruction	12 (7.8%)	10 (6.4%)	
pT			0.717
Is	29 (19%)	24 (15.4%)	
1	74 (47.7%)	74 (47.4%)	
2	47 (30.7%)	56 (35.9%)	
3	3 (2.0%)	2 (1.3%)	
4	1 (0.7%)	0	
pN			0.807
0	114 (74.5%)	111 (71.2%)	
0 (*i*+)	9 (5.9%)	7 (4.5%)	
1	19 (12.4%)	23 (14.7%)	
1 mi	8 (5.2%)	12 (7.7%)	
2	2 (1.3%)	3 (1.9%)	
3	1 (0.7%)	0	

Abbreviations: DCIS: ductal carcinoma in situ; HR: hormone receptor; IDC: invasive ductal carcinoma; ILC: invasive lobular carcinoma.

**FIGURE 2:**
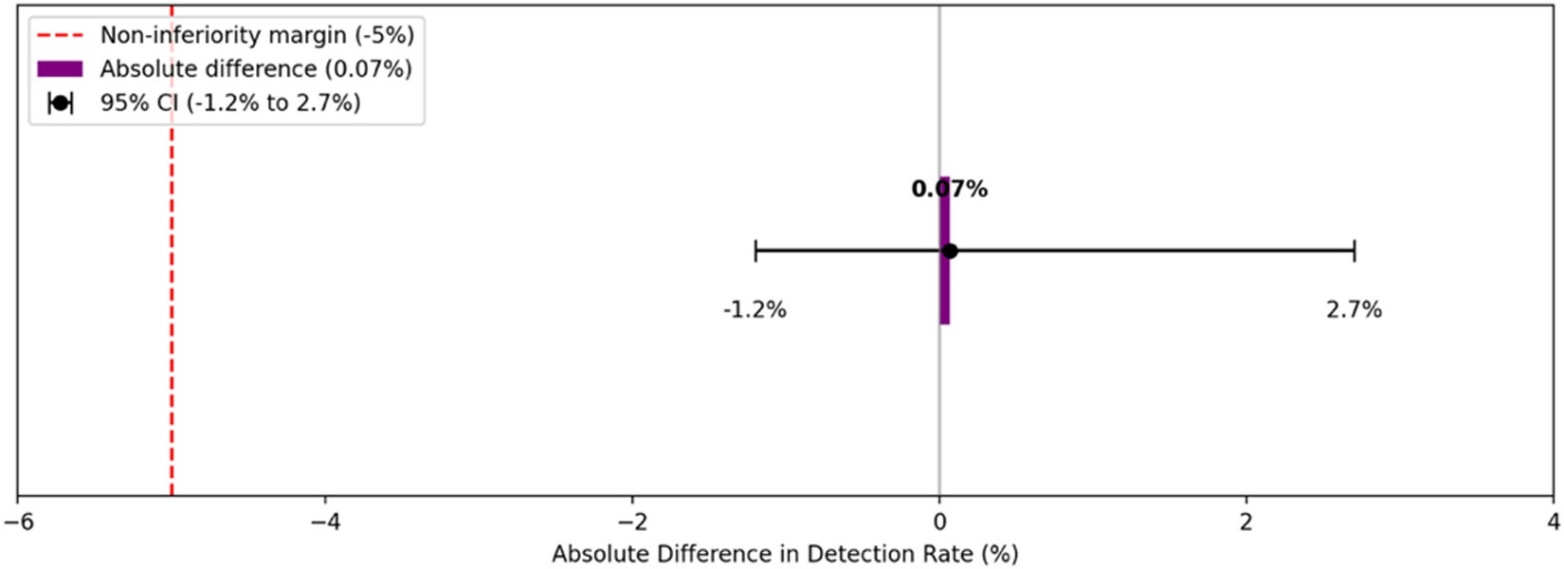
Absolute difference in detection rate between SPIO and dual tracer.

The SLN detection rate was 99.3% (95% CI: 95.8–99.9) in the SPIO arm and 100% (95% CI: 97–99.9) in the dual tracer arm (Table 2). The absolute difference in detection rate was 0.7% (95% CI: −1.2–2.7), meeting the predefined non‐inferiority margin (Figure 2). SLNB failed in one patient in the SPIO group. A 52‐year‐old patient with a BMI of 32 had a T1 retroareolar tumor and was undergoing breast conservative surgery. 1 mL of Magtrace was injected 9 days before surgery at the periareolar region. Preoperatively, 5 cm brownish skin pigmentation was noted over the lower outer quadrant around the areola. Sentinel lymph node localization failed, with minimal probe reading at the axilla and no colored nodes identified. Formal axillary dissection revealed no metastasis.

**TABLE 2:** Operative outcomes.

	SPIO group	Dual tracer group	*p* value
Successful SLNB	152 (99.3%)	156 (100%)	0.495
Identification rate, 95% CI	99.3% (95.8–99.9)	100% (97–99.9)	
Total number of SLN removed	517	412	
Magnetic/Radioactive + colored	485	321	
Colored only	3	49	
Magnetic/Radioactive only	29	42	
Timing of injection (day), median (IQR)	3 (1–7.5)	—	
Positive SLN	21 (13.7%)	20 (12.8%)	0.819
Axillary dissection performed	23 (15%)	22 (14.1%)	0.207
Transcutaneous signal detected	106 (69.3%)	145 (92.9%)	< 0.01
Operating time for SLN (min)	25 (17–35)	22 (14–35)	0.073
Number of SLN, median (IQR)	3 (2–4)	2 (2–4)	< 0.001
Time per SLN, median (IQR)	7.9 (3.9–12.9)	10 (6.7–13.5)	0.036
Complications	26 (17%)	19 (12.2%)	0.23
Seroma	6	6	
Pigmentation	13	9	
Infection	1	2	
Others	6	2	

Operative outcomes are shown in Table 2. The transcutaneous signal detection was lower with SPIO (69.3%) than dual tracer (92.9%; *p <* 0.01). However, after incision, magnetic signal was detected in the axilla in all but one failed case. No additional tracers were injected preoperatively in the SPIO arm. The total number of SLN excised in the SPIO arm was higher (517 vs. 412), with a median of 3 (IQR 2–4) versus 2 (IQR 2–4; *p* < 0.001). Table 3 shows that the SPIO group yielded a higher number of SLN retrieved per procedure, with more cases retrieving more than 3 nodes compared with dual tracer (69 vs. 40; *p* < 0.001), while dual tracer more often yielded 1 to 2 nodes (90 vs. 54; *p* < 0.001). The operating time for SLNB was longer in the SPIO group (25 vs. 22 min; *p* = 0.073). The median time per SLN harvested was significantly shorter in the study group (7.9 vs. 10 min; *p* = 0.036).

**TABLE 3:** Number of sentinel lymph nodes retrieved.

	0 SLN	1 SLN	2 SLN	3 SLN	More than 3 SLN	*p* value
SPIO group	1	18	36	29	69	< 0.001
Dual tracer group	0	34	56	26	40	

Two patients in each group had false negative SLNB. They had successful identification of hot and colored sentinel lymph nodes with residual axillary count < 10%. In addition, non‐sentinel lymph nodes were identified by the operating surgeon as palpably enlarged or hard axillary lymph nodes, these came back to show macrometastasis, and subsequent axillary dissection was performed in the same session. The false negative rate of the SPIO group was 8.7% (2 out of 23) and 9.1% (2 out of 22) for the dual tracer group.

26 patients (17%) in the study group and 19 (12.2%) in the control group experienced complications, with hyperpigmentation present in up to 50% of the patients (brown or blue). Brown hyperpigmentation (SPIO arm) was noted to last up to 36 months post‐operatively in one patient, and 77% still had brown staining at 12 months. The second most commonly seen complication was seroma formation. This was likely due to the relatively high mastectomy rate in both groups (around 80%) However, the difference in complication rate was not statistically significant. No anaphylactic reactions were seen in either group.

In subgroup analysis, binary logistic regression identified younger age (*p* = 0.01) and perioperative SPIO injection (*p* < 0.01) were associated with a higher chance of transcutaneous signal detection in the study group. Tumor location, size, and BMI showed no association. Injection timing (peri‐operative vs. preoperative) did not significantly affect the SLN yield, number of colored nodes, operative time or peak probe counts.

## 4 Discussion

The introduction of SLNB in the 1990s represented a pivotal advancement in the surgical management of early breast cancer, establishing it was the standard of care for axillary staging in clinically node‐negative patients [13]. Conventional techniques, including blue dye alone, Tc‐99m colloid or their combination, have demonstrated SLN identification rates approaching 99% in meta‐analyses [11]. Despite these high performance metrics, the dual‐tracer approach is associated with several limitations. These challenges have prompted the development of alternative lymphatic mapping agents, among which SPIO has emerged as a promising option. Previous cohort studies have demonstrated the comparable SLN detection rate of SPIO to conventional dual tracer method, ranging from 94.4% to 99% [3, 5, 6, 7, 8, 9, 10]. However, many of those trials administered SPIO concurrently with blue dye or radioisotope, which may introduce synergy bias and overestimate the standalone efficacy of the magnetic tracer. In this study, SPIO was used as a single agent and has demonstrated its non‐inferiority, with an identification rate of 99.3%.

The use of SPIO confers several practical advantages over conventional methods, including the absence of ionizing radiation, independence from nuclear medicine department scheduling and a wide therapeutic window for injection (up to 30 days preoperatively). These features enhance surgical flexibility and facilitate out‐patient injections and benefit patients' convenience. It can also be used to minimize axillary surgery in cases with in situ carcinoma, where there may be a chance of pathology upgrade. If pre‐operative SPIO injection was done, only when invasive carcinoma is confirmed, would formal SLNB be performed without the need of injection of the tracer again [14, 15].

Compared with earlier formulations such as Sienna XP (Endomagnetics Ltd., Cambridge, UK), which required dilution, Magtrace is ready to use. Some studies have shown that lower dosages of SPIO showed similar SLN detection rates compared with conventional method (2 mL) and had less skin discoloration [16, 17]. As a result, we lowered the volume of SPIO injected to 1 mL, and it did not affect the SLNB procedure. 77% of the patients with pigmentation post‐operatively had 2 mL SPIO injected. However, this study was not powered to compare the difference in volume of SPIO injected and the degree of skin staining.

The significantly higher median number of SLNs retrieved with SPIO (3 vs. 2; *p* < 0.001) corroborates the finding from a local randomized trial and meta‐analysis [11, 18]. Although the optimal number of SLN harvest is debated, retrieval of additional nodes may enhance staging accuracy, particularly in the neoadjuvant setting, where more than 3 SLNs are associated with false negative rates below 10% [19, 20, 21].

Although total SLNB operative time was longer in the SPIO arm (25 vs. 22 min), this difference did not reach statistical significance (*p* = 0.073). This has also been reported in previous studies, as the use of the SentiMag probe requires repeated recalibration and exchange of surgical instruments to polymer ones [5]. There may also be a learning curve for this new technique compared to the traditional dual tracer in our study centers. Another point to note is that the time per sentinel lymph node retrieved in the SPIO arm was shorter (7.9 vs. 10 min; *p =* 0.036), reflecting accurate axillary staging without prolonging the operation time.

Apart from the identification success of an SLNB procedure, the false negative rate (FNR) is also important as it may lead to understaging and increased risk of cancer recurrence. A systemic review of 69 trials found the FNR of SLNB to be 7.3% (range 0%–29%) [22]. In the neoadjuvant setting, a study showed the FNR of SLNB to be 8.4% for SPIO and 10.9% for dual tracer, which is comparable to our study [20]. This can be reduced by obtaining more than 1 sentinel lymph node [23], which is seen in the SPIO group, where the median number of lymph nodes harvested is higher. Moreover, this reflects the importance of the clinical judgment of the operating surgeon, where gentle palpation of the axilla should be done to identify any suspicious nodes. These suspicious nodes should also be considered sentinel lymph nodes and removed accordingly, although for the purpose of this study, they were considered to be non‐SLN by tracer detection. This may be because tumor involvement of the axillary lymph nodes can cause interference with the uptake of both dual tracer and SPIO, as lymph flow may be diverted to another node rather than the true sentinel node [24].

Discoloration from SPIO can persist up to 15 months after surgery, although becoming smaller and paler with time. An extended duration of staining was strongly associated with breast‐conserving surgery [25]. This was also noted in our study, with the majority of our patients experiencing periareolar pigmentation, having received breast‐conserving surgery. As the tracer solution is injected into the perioareolar region, the area may not be excised in breast‐conserving surgery. Deeper injection at the areola, lower dosage of injection, and peritumoral injections can be considered to lessen the discoloration [7, 9, 14]. Furthermore, persistent magnetic tracer reflected by the discoloration has been shown to cause artifacts in MRI scans, which can last up to 25 months after injection [26, 27]. Although the size and intensity of skin discoloration did not predict the degree of MRI artifacts, this should be a fact to consider in patients where follow‐up breast MRI may be needed for surveillance. Patient counseling regarding these long‐term effects is essential during informed consent.

There are a few limitations in this study. Although this was a multicenter trial, patient accrual was prolonged due to the COVID‐19 pandemic affecting surgery scheduling and manpower shortage in the public sector. A longer study period may allow for better SLNB techniques with SPIO due to a learning curve, which may have been reflected in the shorter time per sentinel lymph node harvested in the study group. Blinding of the surgeons and patients was not possible in this randomized controlled trial. Baseline characteristics showed statistical significance between the two groups in terms of tumor location. Stratification with variables, including tumor location, can be considered during randomization for future studies to address this.

In conclusion, this randomized clinical trial found that SPIO as a single agent is non‐inferior to the conventional dual tracer method in SLNB for early breast cancer patients. These results suggest that SPIO is a good alternative to dual tracer and encourage its widespread use to avoid radioactive isotope difficulties.

## Author Contributions

**Chiu Hoi Woon Tiffany:** data curation, formal analysis, writing – original draft, writing – review and editing, investigation, methodology. **Ray Ka Wai Hung:** conceptualization, data curation, writing – review and editing, project administration, supervision, funding acquisition. **Kong Wai Chung Angela:** data curation, investigation, project administration. **Kwok Sin Man:** data curation, investigation, project administration. **Sheung Robin Lok Man:** data curation, investigation, project administration. **Violet Yee Kei Tsoi:** conceptualization, supervision, project administration, funding acquisition, resources.

## Funding

Magtrace solution was supplied by Endomagnetics Ltd. (Cambridge, UK).

## Disclosure

This study was supported Endomagnetics Ltd. (Cambridge, UK), which provided the Magtrace tracer for the period of the study.

## Consent

Informed consent was obtained from all individual participants included in the study. The study was approved by local ethics committee before commencement.

## Conflicts of Interest

The authors declare no conflicts of interest.

## Data Availability

The data supporting the findings of this study are available within the article. Raw individual participant data are not publicly available due to privacy protections but may be obtained from the corresponding author upon reasonable request, subject to ethics approval and a data use agreement.
